# Unusual Thyroid Nodule: A Case of Symptomatic Thyroid Schwannoma

**DOI:** 10.7759/cureus.11425

**Published:** 2020-11-10

**Authors:** Shaher Abbarah, Shahem Abbarh, Bandar AlHarthi

**Affiliations:** 1 General Surgery, Almaarefa University, Riyadh, SAU; 2 Internal Medicine, Almaarefa University, Riyadh, SAU; 3 Surgery, King Fahad Medical City, Riyadh, SAU

**Keywords:** case report, neck mass, schwannoma, thyroid nodule, recurrent laryngeal nerve

## Abstract

Primary thyroid schwannoma is a sporadic non-epithelial tumor of the thyroid gland. Most previous cases reported in the literature presented it as an asymptomatic neck mass. In this report, we describe a rare case of thyroid schwannoma in a 33-year-old female, presented as a left-side neck swelling, accompanied by a change in voice and difficulty swallowing. An ultrasound of the mass showed a large heterogeneous, predominantly cystic, nodule; in contrast to the usual predominantly solid nodule described in the literature. After several pre-operative diagnostic tests, including fine needle aspiration (FNA), the diagnosis remained unclear. In the end, the patient underwent a successful surgical excision of the mass, and the diagnosis of schwannoma was confirmed based on the histopathologic examination which revealed both type A and B Antoni cells as well as positive staining for S-100 protein.

## Introduction

Schwannomas (also known as neurinomas or neurilemmomas) are rare tumors originating from the Schwann cells of the cranial, peripheral, and autonomic nerve sheaths. These are generally benign, slow-growing tumors with malignant transformation being extremely rare [1]. Schwannomas may develop in any location in the body, with 25 to 45% of extracranial schwannomas appear in the head and neck region [2]. Thyroid involvement constitutes a particularly rare situation. Primary Schwannoma of the thyroid gland was first reported in 1964 by Delaney and Fry [3]. Since then, there have been only a few cases of primary thyroid schwannoma described in the literature. Schwannomas are often asymptomatic; however, various symptoms, such as a change in voice and dysphagia, may appear in occasional cases due to compression of the adjacent structures or direct nerve invasion [4]. Thyroid schwannoma usually presents as a solitary, palpable neck mass [1]. With its rare incidence, the diagnosis can be challenging, and the surgeon should be cautious when dealing with such a case. Herein, we describe an unusual case of symptomatic thyroid schwannoma presenting as a neck mass and associated with a change in voice and difficulty swallowing. Informed consent was obtained from the patient to publish this case report.

## Case presentation

A 33-year-old housewife was referred to our surgical department at King Fahad Medical City, Surgical department from a local primary hospital as a case of a left-side neck mass. The patient noticed a painless, increasing in size, left-side neck swelling five months before the presentation while she was pregnant. She also reported hoarseness and occasional dysphagia, particularly with solid food. Apart from palpitation at exercise, the patient denied any symptoms of hypo- or hyperthyroidism. She is asthmatic and takes albuterol and oral contraceptive pills-no previous surgeries.

Clinical examination revealed a well-defined, approximately 6 x 2 cm in size, non-tender nodule on the left side of the neck, with a smooth surface-the swelling moves with swallowing, and no evidence of retrosternal extension. Cervical and supraclavicular lymph nodes were not palpable. At this stage, the differential diagnosis included thyroid adenoma, malignant thyroid nodule, and non-epithelial tumor of the thyroid gland.

**Figure 1:**
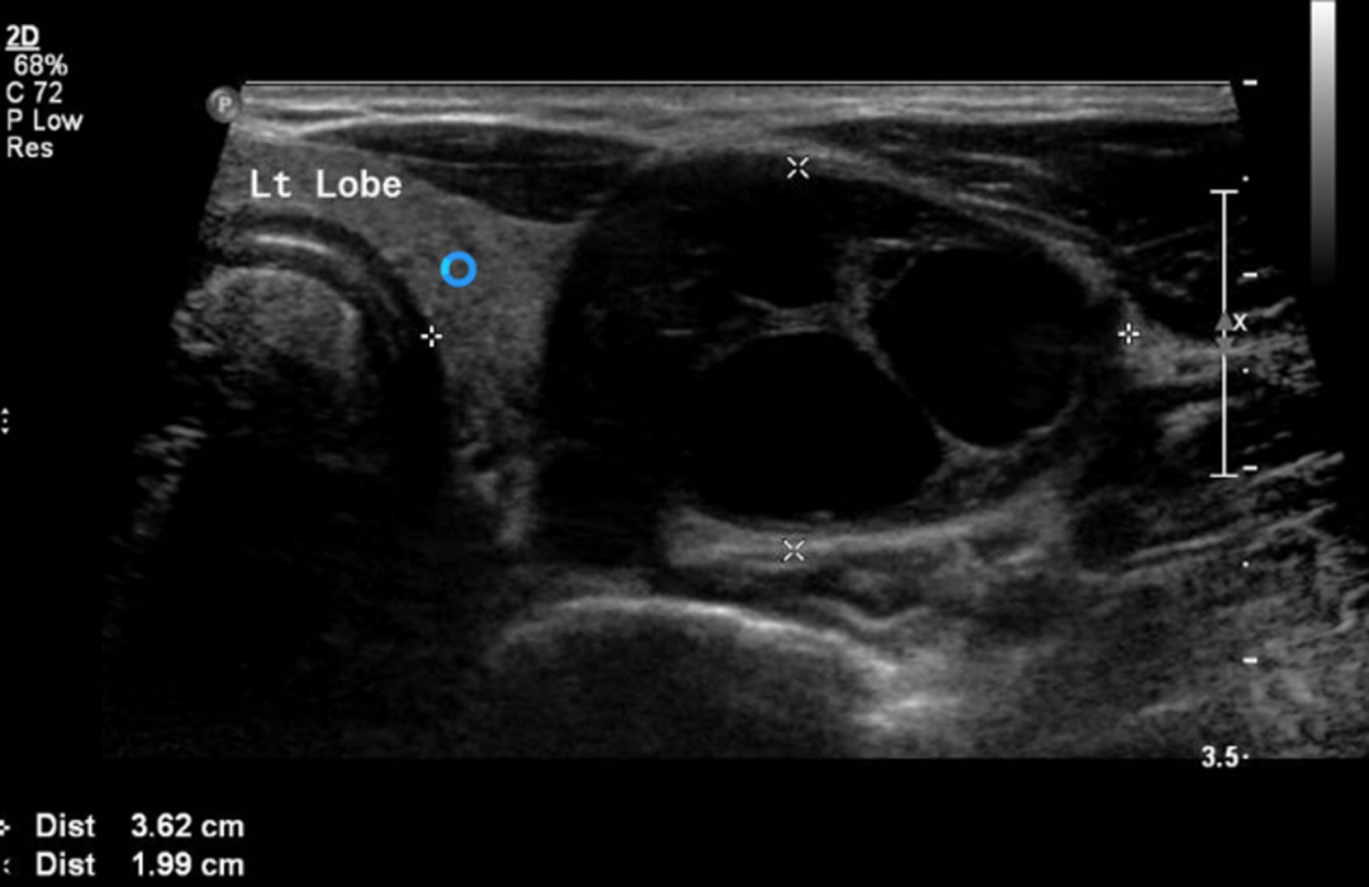
Ultrasonography of the thyroid gland revealing left lobe of 3.6 x 2.0 cm in size with a large heterogeneous, predominantly cystic, nodule measuring about 7.5 x 2.4 cm in the longest dimensions

An initial ultrasound (U/S) of the neck at the local primary hospital showed a well-defined left thyroid lobe, measuring about 5.75 x 2.5 cm, with multiple cystic areas, and marked internal vasculature. Repeated U/S at our hospital revealed 3.6 x 2.0 cm left thyroid lobe, with a large heterogeneous, predominantly cystic, nodule measuring approximately 7.5 x 2.4 cm. No cervical lymphadenopathy was identified (Figure 1).

Two, one month apart, U/S-guided fine needle aspiration (FNA) showed nondiagnostic result with cystic fluid only (Bethesda I) [5]. After six months, a repeated FNA revealed atypia of undetermined significance (Bethesda III); smear was hypocellular with rare colloid material and foci suspicious for ill-defined granuloma formation. Flexible laryngoscope identified left vocal cord paralysis with compensation from the right side, and a minimal gap of 1 mm. Thyroid function tests were done several times and were within normal limits.

**Figure 2:**
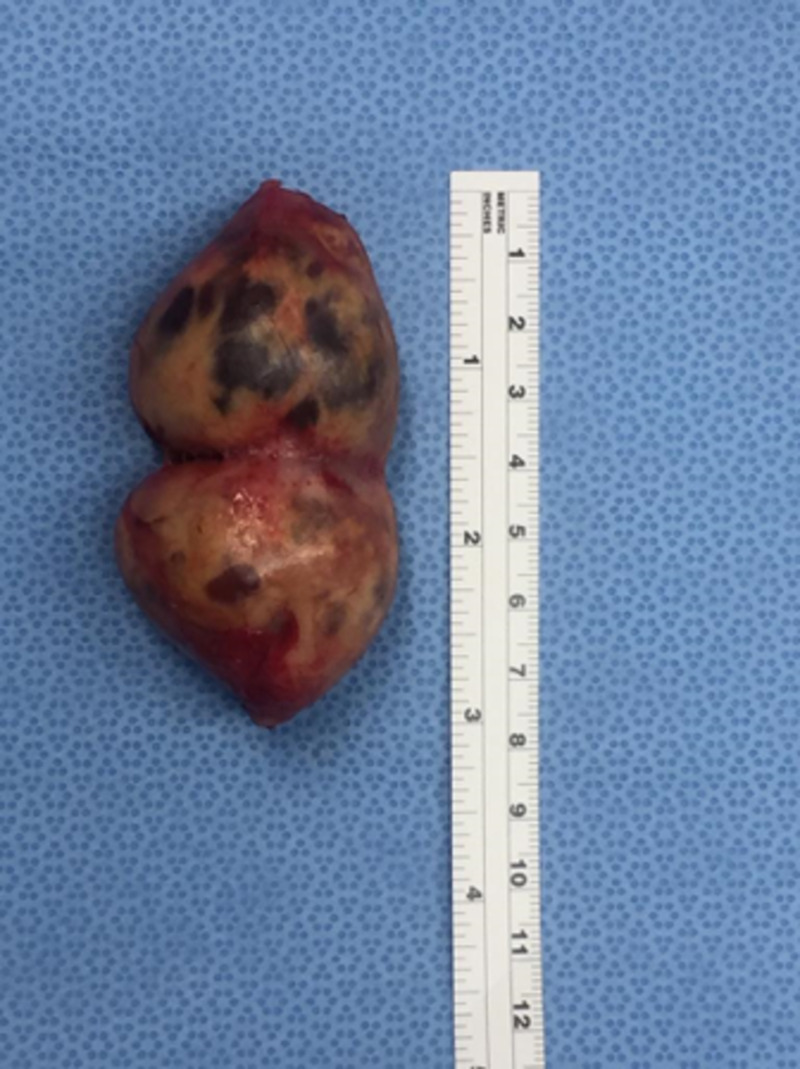
Gross picture of the removed mass after its separation from the thyroid tissue

**Figure 3:**
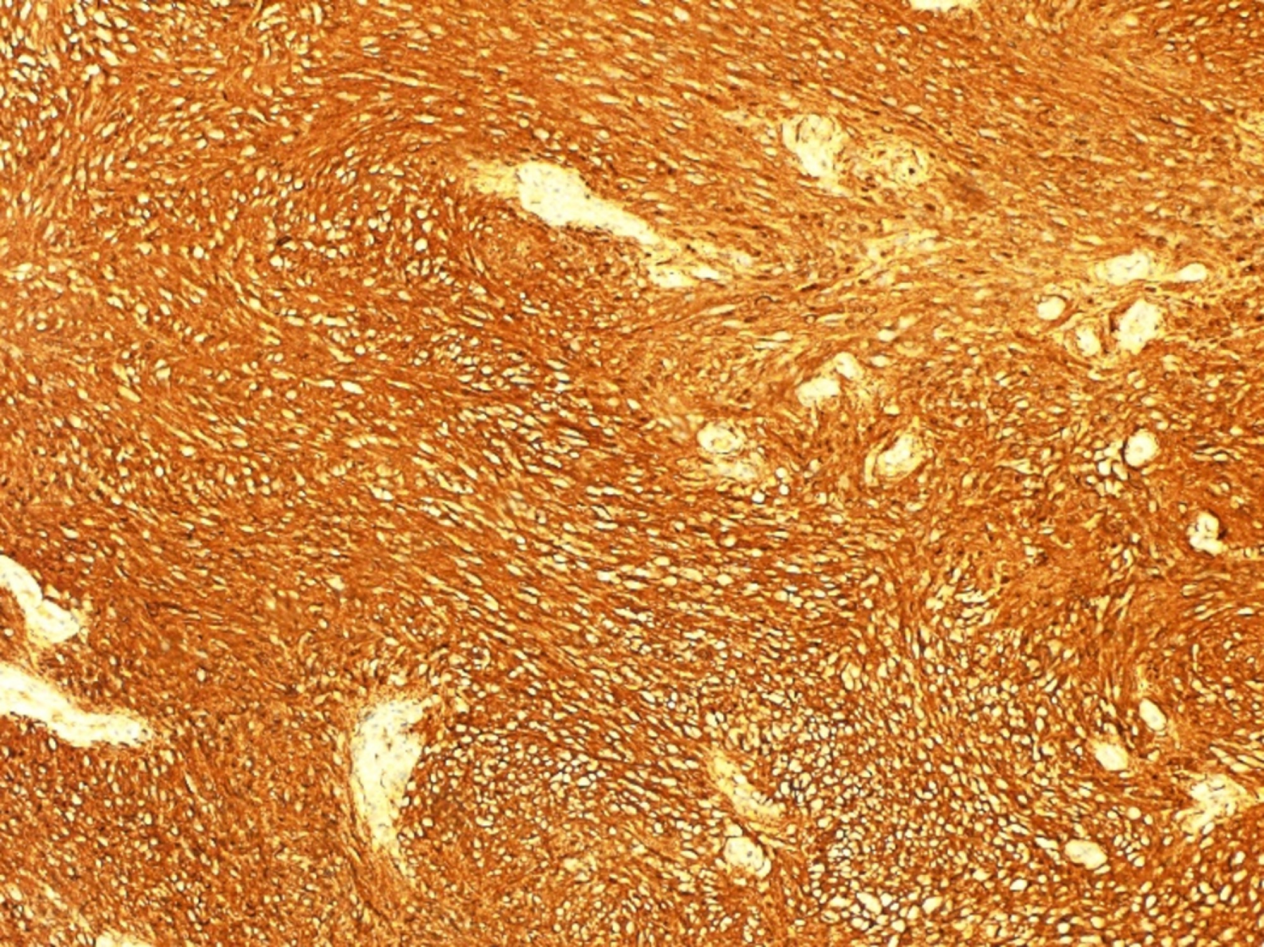
Immunohistochemistry shows diffuse positive staining for the S-100 protein

The patient was admitted electively as a case of left side thyroid mass for surgical excision. Intra-operation: the cystic lesion was identified and dissected laterally to the carotid sheath inferiorly and superiorly. On the upper edge of the cystic lesion, there were feeding vessels which were divided using vicryl suture. After that, the lesion was easily separated from the thyroid tissue (Figure 2) and sent to the histopathology lab, which showed mixed Antoni A and B cells, with strong and diffuse immunohistochemical staining for S-100 protein (Figure 3).

The left thyroid lobe was intact with no other cystic lesions. Post-operation: there were no complications, and the patient did not report any more difficulties in swallowing or voice changes. At one-month follow-up, the patient reported some improvement in her voice, and she was referred to the Otorhinolaryngology department.

## Discussion

Primary non-epithelial tumors of the thyroid gland are sporadic. These could be of vascular, smooth muscle, or neural origin. Thyroid Schwannomas arise from neuronal sheath cells of the vagus nerve and, less commonly, from the sympathetic chain and hypoglossal nerve. Their incidence is rare, with only a few cases have been reported in the English literature. Most of the reported cases were localized in the right lobe, a few involved the left lobe, as in our patient, while only one case was reported involving the isthmus of the thyroid gland [6]. Aoki et al. suggested that neurologic asymmetry of the thyroid gland may play a role in this right lobe predilection of schwannoma [7].

Most thyroid neurilemmomas present as a neck mass in an otherwise asymptomatic patient, though local neck symptoms related to compression or invasion of the adjacent structures may appear. In the current patient, the left recurrent laryngeal nerve was involved causing progressive hoarseness and dysphagia. Similarly, hoarseness and dysphagia have been reported in one patient with schwannoma originating from the left thyroid lobe [8], as well as one another case with isthmus involvement [6]. Less often, symptoms have also been reported in the literature; one patient presented with a complaint of the flushed face and easy fatigability [9]. Another patient with a large tumor of the right lobe extending as far as the retrosternal space presented with dyspnea due to tracheal compression [10].

Schwannomas are often challenging to diagnose and require several diagnostic modalities. U/S imaging is often ordered in the workup of thyroid masses. Sonographic findings usually describe schwannoma as a well-defined, round or ovoid, hypoechogenic predominantly solid nodule [11,12]; on the contrary, our case presented with the predominantly cystic nodule. Another diagnostic tool is FNA which is readily available and accurate for most head and neck masses. However, FNA findings in schwannoma cases are often inconclusive. In a case series of thirty patients with extracranial head and neck schwannoma, the accuracy of FNA was only 20% [13]. In the same line, our patient had a total of three U/S-guided FNA, and all were inconclusive. Interestingly, thyroid hormones levels are generally within the normal range, as is the case in our patient.

Additional studies that might be considered for assessment of a neck mass include Computed Tomography (CT) and Magnetic Resonance Imaging (MRI) of the head and neck area. The CT appearance of thyroid schwannoma is usually a well-circumscribed homogeneous mass of soft tissue density, though non-homogeneous masses have been reported as well [9]. A target sign on the Magnetic Resonance Imaging (MRI) has 100% specificity and 59% sensitivity [14]. When Tc-99m pertechnetate thyroid scan was performed in previous cases, it always showed a cold nodule [6,15,16]. Hence, when evaluating a cold nodule of the thyroid gland, neurogenic tumors should be considered.

After all, given the rare incidence of thyroid schwannoma, along with its unspecific clinical features and the undiagnostic findings in most of the workup performed, pre-operative diagnosis of thyroid schwannoma is often difficult. Instead, diagnosis is often made post-operative based on histopathological examination of a surgical specimen. Histologically, neurilemmomas are classified into two types: Antoni A (spindle‑shaped Schwann cells with nuclear palisading) and Antoni B (pauci-cellular areas with myxoid change) [17]. The majority of the reported cases, including our case, exhibit both Antoni A and B characteristics. Furthermore, positive S-100 immuno-staining supports the diagnosis of schwannoma and aids in distinguishing it from other mesenchymal tumors.

Currently, the usual treatment for thyroid schwannoma consists of surgical resection, which is considered to be curative. Generally, a lobectomy or mass excision is performed with an excellent prognosis, low post-operative complications, and low risk for tumor recurrence. The current patient underwent surgical removal of the mass successfully.

## Conclusions

Primary schwannomas of the thyroid gland are sporadic, and the few documented cases in the literature differ in the clinical course, presenting symptoms, imaging features, and FNA findings. Despite their rarity, schwannomas should be taken into consideration in the differential diagnosis of thyroid nodules, particularly a single cold thyroid nodule. Complete surgical resection is considered to be a curative therapeutic strategy and appears to be the treatment of choice.
